# Rapid Detection and Differentiation of *Clonorchis sinensis* and *Opisthorchis viverrini* Using Real-Time PCR and High Resolution Melting Analysis

**DOI:** 10.1155/2014/893981

**Published:** 2014-10-19

**Authors:** Xian-Quan Cai, Hai-Qiong Yu, Rong Li, Qiao-Yun Yue, Guo-Hua Liu, Jian-Shan Bai, Yan Deng, De-Yi Qiu, Xing-Quan Zhu

**Affiliations:** ^1^State Key Laboratory of Veterinary Etiological Biology, Key Laboratory of Veterinary Parasitology of Gansu Province, Lanzhou Veterinary Research Institute, Chinese Academy of Agricultural Sciences, Lanzhou, Gansu Province 730046, China; ^2^Technical Center, Zhongshan Entry-Exit Inspection and Quarantine Bureau, Zhongshan, Guangdong Province 528403, China; ^3^Technical Center, Guangdong Entry-Exit Inspection and Quarantine Bureau, Guangzhou, Guangdong Province 510630, China; ^4^Guangzhou Airport Entry-Exit Inspection and Quarantine Bureau, Guangzhou, Guangdong Province 510470, China

## Abstract

*Clonorchis sinensis* and* Opisthorchis viverrini* are both important fish-borne pathogens, causing serious public health problem in Asia. The present study developed an assay integrating real-time PCR and high resolution melting (HRM) analysis for the specific detection and rapid identification of* C. sinensis* and* O. viverrini*. Primers targeting COX1 gene were highly specific for these liver flukes, as evidenced by the negative amplification of closely related trematodes. Assays using genomic DNA extracted from the two flukes yielded specific amplification and their identity was confirmed by sequencing, having the accuracy of 100% in reference to conventional methods. The assay was proved to be highly sensitive with a detection limit below 1 pg of purified genomic DNA, 5 EPG, or 1 metacercaria of* C. sinensis*. Moreover,* C. sinensis *and* O. viverrini* were able to be differentiated by their HRM profiles. The method can reduce labor of microscopic examination and the contamination of agarose electrophoresis. Moreover, it can differentiate these two flukes which are difficult to be distinguished using other methods. The established method provides an alternative tool for rapid, simple, and duplex detection of* C. sinensis *and* O. viverrini*.

## 1. Introduction

*Opisthorchis viverrini* and* Clonorchis sinensis* are pathologically important members of the family Opisthorchiidae. Chronic infections with these liver flukes closely associate with the development of the bile duct cancer (cholangiocarcinoma) and the liver cancer (hepatocarcinoma) in humans [1]. In a recent survey in Chinese endemic areas, the overall prevalence of* C. sinensis *infection was 0.58% in 356,629 residents from 688 sampled pilot sites in China [2]. Opisthorchiasis caused by* O. viverrini* is considered an important food-borne parasitic disease in Southeast Asia including Thailand, Lao PDR, Vietnam, and Cambodia; approximately 67.3 million people are at risk of the infection [3]. Humans get infected by ingestion of undercooked or pickled freshwater fish containing metacercariae of* O. viverrini* and* C. sinensis*, which are classified by the International Agency for Research on Cancer as a group 1 biological carcinogen in 2009 [4].

*C. sinensis* and* O. viverrini* have similar life cycles, locations, pathogenicity and their morphologies, in particular the forms of their eggs and metacercariae differ only slightly [5]. However, the ability to differentiate the species of liver and minute intestinal flukes is important from both a clinical and epidemiological perspective. The frequency and types of pathology and clinical diseases among* C. sinensis* and* O. viverrini *seem to differ; for example, cholelithiasis is one of the most serious complications of clonorchiasis but a rare complication of opisthorchiasis [6]. Although both flukes are implicated as predisposing factors for cholangiocarcinoma, this is more frequent with* O. viverrini.* From an epidemiological perspective,* C. sinensis* will make control more challenging.

A number of methods have been developed to detect these two species, such as direct microscopy, ELISA [7], conventional PCR [8], and loop-mediated isothermal amplification (LAMP) [9]. There are, however, many drawbacks of these methods. For example, microscopic examination is cumbersome and time consuming, requiring experienced laboratory technicians, and often it is difficult to distinguish eggs from those of closely related heterophyids, which have similar egg morphologies. Eggs can therefore only be characterised as “*Opisthorchis/Clonorchis*-like” eggs [10]. Crude or recombinant antigens have been evaluated for immunodiagnosis; however, the specificity and sensitivity of these methods are still in great need of improvement [11, 12]. Both conventional PCR and LAMP have high risk of contamination. Moreover, even with expensive probe labeled real-time PCR, the two liver flukes are difficult to be distinguished [13].

Recently, high resolution melting (HRM) for fast, high-throughput analysis of many pathogens has been developed, for example, variation scanning [14], species determination [15], genotyping [16], and even the identification of recent and nonrecent HIV infections [17]. The real-time PCR platform with HRM is a single-step closed tube, reduces turnaround time of the assay reported here to almost 1 h, eliminates the risk of contamination, and saves expense. These features make it advantageous for use in laboratories. Here, we developed a real-time PCR assay coupled with HRM analysis for rapid identification and differentiation of* C. sinensis *and* O. viverrini*.

## 2. Materials and Methods 

### 2.1. Samples

Adult worms of* C. sinensis* were collected from naturally infected cats in Guangdong Province. Adult worms of* O. viverrini* were kindly provided by Professor Sung-Jong Hong, Department of Medical Environmental Biology, Chung-Ang University College of Medicine, Republic of Korea, which were originated from the Lao PDR. Several closely related trematodes infecting humans were included as “heterologous” control samples for assessing the specificity of the real-time PCR assay, namely,* Fasciola hepatica, Fasciola gigantica, Schistosoma mansoni, *and* Schistosoma japonicum.* All parasite materials were preserved in 70% ethanol and kept at −20°C until the extraction of genomic DNA. Metacercariae of* C. sinensis* were collected from the fish muscles essentially according to a previously reported method [9]. Fecal samples were obtained from the residents in Guangdong Province, China. The fecal samples were examined three times by the Kato-Katz thick smear using nylon screens and plastic templates.

### 2.2. Genomic DNA Extraction

Total genomic DNA was extracted from adult worms, using the commercial QiAamp DNA extraction kit (Qiagen). In the case of adult worms, only a single specimen was used in each DNA extraction. Briefly, the sample was enzymatically digested in 180 *μ*L of a lysis solution (ATL buffer-Qiagen); then 20 *μ*L proteinase-K (50 g/mL) was added and incubated at 56°C for 2-3 h with brief vortexing every 30 min. For eggs, the incubation time was set 1-2 h longer. The mixture, after adding 200 *μ*L buffer AL (Qiagen) containing guanidine hydrochloride and 4 *μ*L RNase A (100 mg/mL) and mixing by pulse-vortexing for 15 s, was further incubated at 70°C for 10 min. Thereafter, 200 *μ*L of ethanol (96–100%) was added and mixed by vortexing for 15–20 s. The contents were then loaded onto a QIAamp Spin Column for DNA binding and spin down for 1 min. The column with the DNA bound was washed several times using solutions (AW1; AW2 buffers—Qiagen) provided according to manufacturer's instruction. Finally, the genomic DNA was eluted in 50 *μ*L elution buffer (AE) and stored at −20°C until use. The DNA from fecal samples was extracted using QIAamp DNA Stool Mini Kit according to the manufacturer's instructions, and the DNA from metacercariae in fish tissue was extracted according to a previous description [9, 13]. The concentration of DNA samples was estimated using Thermo Nanodrop 1000.

### 2.3. Primers, Real-Time PCR, and HRM Analysis

A pair of primers targeting the COX1 gene of* C. sinensis* and* O. viverrini* (COX1e-F: 5′-GGTAGGGTGGTTTGAGC-3′ and COX1e-R: 5′-TCATAGTAACCGAGCTAAA-3′) was synthesized by Takara Biotechnology (Dalian, China). Real-time PCR amplification was performed in Lightcycler 480 (Roche, USA). The total reaction volume was 20 *μ*L, which consisted of 1x Fast-Plus EvaGreen qPCR Master Mix (Bio-rad, USA), reaction buffer containing dNTPs, MgCl_2_, fast-activating chemically modified hot start enzyme, Cheetah Taq, 0.3 *μ*M of each primer, and 1 *μ*L genomic DNA as template.

The PCR was carried out with initiation at 95°C for 60 s, then 45 cycles of denaturation at 95°C for 10 s, annealing at 59°C for 10 s, and extension at 72°C for 20 s. When PCR amplification was completed, HRM analysis was performed by lowering the temperature to 60°C for 5 min, followed by increasing the temperature ramping from 60° to 95° at 0.11°C/s, 25 acquisitions/°C. In this process, the PCR amplicons were allowed to denature and reanneal in fluorescence with changes in temperature (*dF*/*dT*). The HRM profile was then analyzed using HRM analysis software, as reported previously [15]. The normalized melting curves were analyzed and clustered samples into different groups with similar melting profiles. Hence, the difference in the shape of the “normalized melting curve” will help cluster samples into different species, and the samples can be detected and grouped at the same time.

## 3. Results

### 3.1. Specificity of the Detection Assay and Confirmation of Amplicon Identity

**Figure 1:**
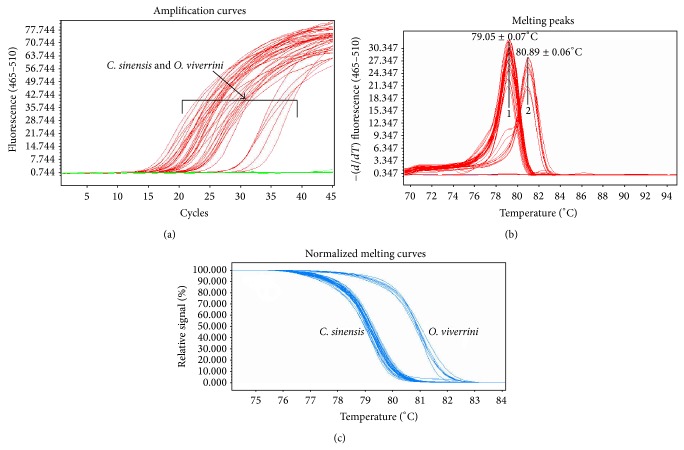
Real-time PCR and HRM analysis for all samples. (a) Real-time PCR amplification for all samples. All the heterologous and blank control had no amplification curve before 40 cycles. (b) Melting peaks of liver flukes 1:* C. sinensis T*_*m*_, 79.05 ± 0.07°C; 2:* O. viverrini*, *T*_*m*_, 80.89 ± 0.06°C. (c) Normalized melting curves for two liver flukes.

The specificity of the primers was determined by performing PCR using pure genomic DNA from* F. hepatica, F. gigantica, S. mansoni, *and* S. japonicum. *A BLAST search of the chosen primer and probe sequences resulted in a hit of the target sequence in* C. sinensis *and* O. viverrini*, suggesting the specificity of the primers. Moreover, no fluorescence signal was detected from the “heterologous control samples,” as mentioned above. In all, 57* C. sinensis* samples, including 36 worms, 11 fish samples, 10 fecal samples, and 10* O. viverrini* worms, get positive results, which was confirmed by microscopic method, while 31 uninfected fish or fecal samples appear negative (Figure 1(a)). To ensure the accuracy of the method, all amplifications were sequenced and proved highly homologous to corresponding sequences.

### 3.2. Detection Limit and Correlation between Cp and Diluted DNA

**Figure 2:**
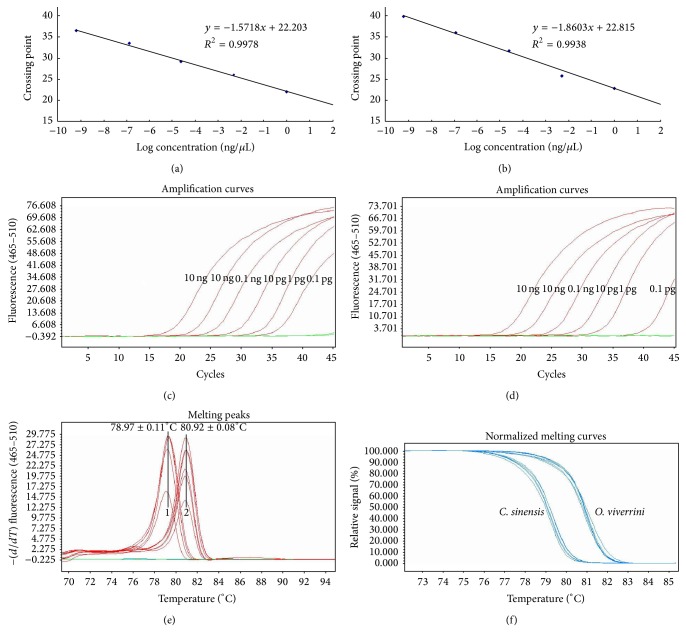
Real-time PCR and HRM analysis of serial diluted genomic DNA. (a) A linear regression of the data providing a formula of *y* = −1.8603log⁡⁡(*x*) + 22.815  (*R* ^2^ = 0.9978), template dilutions 10 ng/*μ*L to 1 pg/*μ*L of* C. sinensis*. (b) A linear regression of the data providing a formula of *y* = −1.5718log⁡⁡(*x*) + 22.203  (*R* ^2^ = 0.9938), template dilutions 10 ng/*μ*L to 1 pg/*μ*L of* O. viverrini*. (c) Amplification curves of diluted genomic DNA of* C. sinensis;* 1–6: dilutions 10 ng/*μ*L to 1 pg/*μ*L. (d) Amplification curves of diluted genomic DNA of* O. viverrini*; 1–6: dilutions 10 ng/*μ*L to 1 pg/*μ*L. (e) Melting peaks of amplicon of diluted genomic DNA from two liver flukes (10 ng/*μ*L to 1 pg/*μ*L); 1:* C. sinensis*, 78.97 ± 0.11°C; 2:* O. viverrini*, 80.92 ± 0.08°C. (f) Normalized melting curves of diluted genomic DNA from two liver flukes (10 ng/*μ*L to 1 pg/*μ*L).

In order to evaluate the detection limit and correlation of the real-time PCR assay, the 10-fold serial dilutions of a genomic DNA were prepared. The DNA was extracted using QiAamp DNA extraction kit (Qiagen) and quantified by spectrophotometry. The 10-fold diluted DNA was performed and good result acquired. The formula of Cp value and template concentration (10 ng/*μ*L to 1 pg/*μ*L) is *y* = −1.8603 log⁡(*x*) + 22.815(*R* ^2^ = 0.9978), when tested with* C. sinensis* (Figure 2(a)), while it is *y* = −1.5718 log⁡(*x*) + 22.203(*R* ^2^ = 0.9938), when tested with* O. viverrini *(Figure 2(b)).

It seems that the sensitivity of* O. viverrini* approaches the sensitivity of* C. sinensis*, which are shown in Figures 2(c) and 2(d). The detection limit of the assay was 1 pg for* O. viverrini* genomic DNA, when considering 40 cycles as the cutoff. To determine the detection limit in fecal samples and fish tissue, 1, 2, 5, and 10* C. sinensis *eggs or metacercariae were spiked into samples, which were proved negative by microscopic method. Each mixed sample was separately used for DNA extraction as described earlier. Finally, we concluded that the detection limit was 5 EPG for fecal samples and 1 MPG (metacercariae per gram fish filet) for fish samples, which was consistent with that of a previous study [13].

### 3.3. HRM Analysis

The sequence difference between the forward and reverse primers will bring different melting temperature (*T*_*m*_) values and normalized melting curves. Constant HRM profiles with distinct *T*_*m*_ peaks were persistently obtained for all positive samples. As shown in Figure 1(b), there were two kinds of characteristic profiles. The amplification product from all 57* C. sinensis* samples had a *T*_*m*_ of 78.05 ± 0.07°C, while the amplification product from 10* O. viverrini* samples had an average 80.89 ± 0.06°C. As shown in Figure 1(c), there were two obvious groups: one represented* C. sinensis* and the other represented* O. viverrini*. To ensure the accuracy of the method, the amplified products were sequenced in both directions. The results showed that all sequences were uniform with the HRM analysis result.

The HRM analysis with different template concentrations (10 ng/*μ*L to 1 pg/*μ*L) of the template appeared to be reliable, while the profile is unsatisfactory when the template concentrations were ≤1 pg. The melting peaks were obtained from each dilution (10 ng/*μ*L to 1 pg/*μ*L), and it shows that *T*_*m*_ of* C. sinensis* was 78.97 ± 0.11, while *T*_*m*_ of* O. viverrini* was 80.92 ± 0.08°C (Figure 2(e)). All the dilutions were divided into two obvious clusters: one represented differently diluted* C. sinensis* and the other represented* O. viverrini *(Figure 2(f)).

## 4. Discussion 

Diagnosis of trematode infection is very important for effective treatment and controlling the spread of infections. Since more and more travelers visited endemic countries, an accurate differential diagnostic method for human clonorchiasis and opisthorchiasis should be developed [1, 2]. Here, we developed an unlabelled real-time PCR with HRM assay targeting the COX1 for the two important liver flukes. The differentiation may not be that important in areas where only one species of the liver flukes is prevalent, but it is necessary in endemic areas where* C. sinensis *and* O. viverrini *are present. Accurate laboratory tests will help diagnose suspected patients.

The method established in the present study has an accuracy of 100% in reference to microscopic methods. Moreover, the detection limit of the method was below 1 pg of purified genomic DNA, 5 EPG, or 1 metacercaria, which was equal to probed real-time PCR [13] and superior to conventional PCR [8]. Real-time PCR using SYBR Green has also been developed targeting many pathogens, but it can only detect gross differences between amplicons generated by real-time PCR, with nonsaturating dyes which would inhibit the PCR reaction. EvaGreen has been proven to be superior to SYBR Green with high reaction efficiencies [18]. The instrumentation for HRM analysis has also been improved with a high rate of data acquisition, ideal optics, tight temperature control, and adequate analysis software; the accuracy of the dissociation* versus* temperature (i.e., melting) curve is as sensitive as 0.01°C, so single base can also be differentiated [19].

*O. viverrini, C. sinensis, *and* Opisthorchis felineus* are the three most medically important species in the family Opisthorchiidae [3]. Now, we still could not validate the detection for the three liver flukes in the same time, because unfortunately both the samples and the genetic information of* O. felineus* are absent. However, once the sequence difference between three species was proved enough, a multiplex real-time PCR for three liver flukes will be developed in the near future.

A previous molecular method targeting the second internal transcribed spacer (ITS2) of rDNA showed good sensitivity, but* O. viverrini* and* C. sinensis* were not able to be differentiated because of high homology [20]. Here, we have designed more than 20 pairs of primers targeting different genes, and the result shows that COX1 is superior to 16S rRNA and ITS rDNA, when used for HRM analysis. Another advantage of mitochondrial DNA (mtDNA) rather than nuclear rDNA is that the mitochondrial genome is present in hundreds or thousands of copies per cell. A further research shows that the length of amplicons smaller than 100 bp is preferable, because shorter amplicon size increases the difference in signal. All these conclusions are in accordance with previous research.

## 5. Conclusion

The present study established a method based on real-time PCR and HRM analysis for the rapid identification and differentiation of* C. sinensis *and* O. viverrini* simultaneously. It offers a potential means for detection of these flukes in the intermediate hosts. Moreover, it would also help in determining the worm burden in fish or infected individuals in endemic areas and would therefore be a useful tool for epidemiological surveys.
